# Laboratory Evaluation of Railroad Crosslevel Tilt Sensing Using Electrical Time Domain Reflectometry

**DOI:** 10.3390/s20164470

**Published:** 2020-08-10

**Authors:** Sijia Li, Chi-Lin Chen, Kenneth J. Loh

**Affiliations:** 1 Department of Structural Engineering, University of California San Diego, La Jolla, CA 92093-0085, USA; sil024@eng.ucsd.edu; 2 Active, Responsive, Multifunctional, and Ordered-Materials Research (ARMOR) Laboratory, University of California San Diego, La Jolla, CA 92093-0085, USA; b04502044@ntu.edu.tw; 3 Department of Mechanical Engineering, National Taiwan University, Taipei 10617, Taiwan

**Keywords:** capacitive, electrical time domain reflectometry, railroad, sensor, structural health monitoring, tilt, track

## Abstract

Crosslevel is defined as the difference in elevation between the top surface of two railroad tracks. Severe changes in crosslevel, for example, due to earthquakes, ground settlement, or crushed ballasts, affect track geometry and can cause train derailment. Therefore, the objective of this study was to monitoring railroad crosslevel by using electrical time domain reflectometry (ETDR) to simultaneously interrogate multiple capacitive tilt sensor prototypes connected in a transmission line. ETDR works by propagating an electrical pulse signal from one end of the transmission line and then monitoring the characteristics of each reflected pulse, which is affected by the capacitance (or tilt) of the sensors. This study begins with a discussion of the capacitive tilt sensor’s design. These 3D-printed sensors were tested to characterize their tilt sensing performance. Then, multiple tilt sensors were connected in a transmission line and interrogated by ETDR. The ability to use ETDR to multiplex and interrogate sensors subjected to different angles of tilt was validated.

## 1. Introduction

In the United States (US) and around the world, railroads are commonly used for transporting goods and people across distant locations. According to the US Department of Transportation, the freight rail network in the US is considered one of the most dynamic systems in the world [1]. It is a $60 billion industry with 140,000 miles of rail tracks, 21 regional railroads, and 510 local railroads [2]. Not only does the US rail network move more freight than any other freight rail system in the world, but it also provides 221,000 jobs across the entire country [1]. However, its heavy usage, as well as exposure to extreme weather and conditions, mean that damage can develop and accumulate in tracks, thereby jeopardizing operations and public safety. In 2019 alone, there were 1848 train accidents in the US according to the US Federal Railroad Administration, of which 1283 were derailments [3]. A notable railroad catastrophe was the derailment that occurred in Washington DC on 18 December 2017. All 12 train cars derailed, which caused three fatalities and approximately 100 injured [4].

While some of these railroad accidents are due to operator-related failures, many train derailments can also be linked to track-related issues, such as being due to broken rails and welds, track geometry, wide gauges, and buckled tracks [5]. First, broken rails and welds can be caused by excessive applied loads, defects from manufacturing, incorrect installation, and extreme weathering. Second, track geometry can change due to environmental effects and ground settlement, as well as extreme loads such as earthquakes. Third, wide gauges, which often occur on the taller side of a curved railroad segment, can cause wheels to drop into the gauge, thus causing a train to derail. Last, extreme events, such as earthquakes and severe temperature variations, can result in excessive compressive stresses that cause tracks to buckle. Therefore, structural health monitoring (SHM) and nondestructive evaluation (NDE) methods are direly needed for detecting these failure events in railroad tracks so that appropriate interventions can be implemented (e.g., halting train operations) for preventing train derailments.

Among these different damage mechanisms, broken rails and welds are the most common causes of train derailments. Common methods used to detect broken rails include using ultrasonic waves and infrared thermography. Ultrasonic inspection of the rail is performed by propagating a Rayleigh wave through the track and then measuring the reflected wave characteristics. The measured reflected ultrasonic wave and its features can allow one to detect the existence of a crack or separation (e.g., by observing changes in time-of-flight measurements). Ultrasonic methods are also commonly used for monitoring stresses in rails, for example, due to temperature effects [6], where the wave speed changes depending on stress. On the other hand, Lo et al. [7] developed a surface sensor to measure the hysteresis loop and Barkhausen effect signal for quantifying stresses in magnetic materials, such as steel railroad tracks. The data acquired was converted into an image that shows the spatial variations in magnetic properties of the structure, whereby stress variations and patterns can be derived from these magnetic properties. In contrast, infrared thermography uses specialized cameras that can convert heat into a thermal image [8]. Cooling of discontinuities in the structure (e.g., an internal crack) can occur at different rates, which results in a nonuniform thermal image that highlight the presence of potential damage. Although these methods can detect damage in railroad tracks, they are limited to near-surface damage features, assume uniform initial heating, and can be time-consuming.

**Figure 1:**
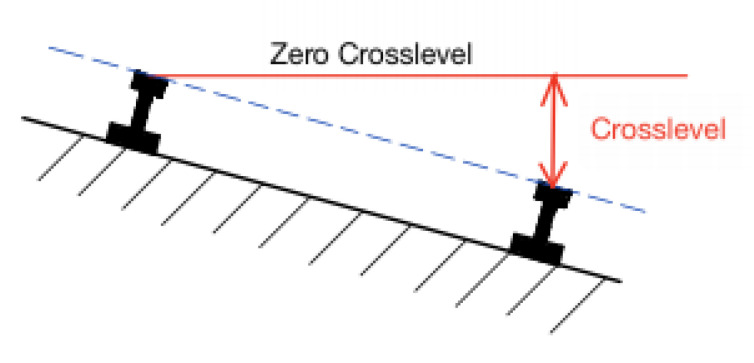
Crosslevel variation between two rails is illustrated.

On the other hand, track geometry changes are the second leading cause of train derailments. The dominant railroad consists of flat-bottom steel rails supported on timber or pre-stressed concrete ties, which rest on crushed stone ballasts. These ballasts permit free drainage and adjustment of tie positions. However, the mobility of crushed stone ballasts also allows ties and rails to undesirably shift their position, thereby leading to track geometry changes or failures. Track geometry is also affected by ground elevation changes induced by environmental variations, such as ground settlement and earthquakes. Track changes are quantified by railroad track crosslevel, which measures the difference in elevation between the top surface of the two tracks (Figure 1). In practice, crosslevel is measured by a bubble-type level, which is filled with water (with a bubble) and can roll along the railroad tracks [9]. The position of the bubble is at the center of the trolley if the elevation of the two tracks is the same, while any changes in track level (or crosslevel) will cause the bubble to shift towards the higher-elevation side. Although this method is simple and accurate, the bubble trolley can only be used when the track is not being operated. Continuous and simultaneous monitoring at multiple locations is also not possible.

As a result of these limitations, many studies investigated alternatives and more advanced means of monitoring railroad track health. An example is the use of fiber Bragg gratings (FBG). In short, FBG is an optical fiber waveguide, and the wavelength of light that is reflected depends on the spacing of periodic variations or modulation of the refractive index within the fiber core [10]. Mi et al. [11] showed that railroad tracks can be monitored using six FBG stress and two temperature sensors installed along a 9 m portion of the track. A different example is a built-in railroad SHM system using piezoelectric lead zirconate titanate (PZT) sensors, as discussed by Park et al. [12]. The system consisted of two PZT patches operated in pitch-catch mode, with one patch used for sending and the other for receiving propagating ultrasonic waves. It was shown that holes and transverse cuts in rails could be successfully detected [12]. While these methods are promising for characterizing defects and residual stresses in individual rails, crosslevel variations between two parallel tracks cannot be monitored using these techniques.

Thus, the objective of this study is to design and validate a capacitive tilt sensor for railroad crosslevel monitoring. As compared to other tilt sensors, such as electrolyte conductors, optics inductance, and resistive mercury balls, capacitive sensors are less sensitive to temperature, humidity, and mechanical misalignment issues [13]. In addition, this study investigated the use of electrical time domain reflectometry (ETDR) for simultaneously interrogating multiple capacitive tilt sensors connected in a transmission line setup. Unlike conventional sensors that each require a separate data acquisition channel and associated tethered cables (which adds significant labor and costs) [10], ETDR interrogates multiple sensors using a single excitation-measurement location [14], similar to an FBG system. This study begins with a theoretical overview of ETDR. Next, the design and fabrication of the capacitive tilt sensors are discussed. Then, the experimental details of characterizing the tilt sensor’s performance and validation of ETDR tilt sensing are presented, followed by a discussion of the results obtained. The manuscript ends with a brief summary of the major findings.

## 2. Electrical Time Domain Reflectometry Background

**Figure 2:**
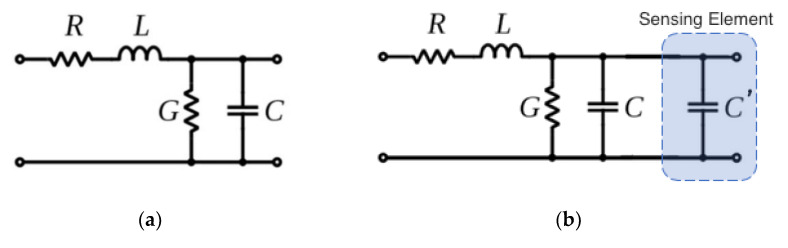
The equivalent circuit models of (**a**) a unit length transmission line and (**b**) a transmission line connected to the capacitive tilt sensor are illustrated.

ETDR is a specific class of time-domain reflectometry methods based on propagating a high-speed electrical pulse waveform at one end of a transmission line and then detecting the characteristics of the reflected wave [15]. In general, a transmission line is an electrical conductor designed to propagate electromagnetic waves or signals [16]. It consists of an inner conductor, outer conductor, dielectric cylinder between the conductors, and a protective jacket [17]. The equivalent circuit model of a unit length transmission line is shown in Figure 2a [18]. The distributed electrical characteristics of a transmission line are described by its series resistance (*R*), series inductance (*L*), shunt conductance (*G*), and shunt capacitance (*C*) [19]. The characteristic impedance (*Z*) of the transmission line can be calculated using this simplified expression [20]:

(1) $$Z=\sqrt{\frac{R+j\omega L}{G+j\omega C}}\approx \sqrt{\frac{L}{C}}$$

where *j* is the imaginary number, *ω* is angular frequency calculated by *ω* = 2*πf*, and *f* is the frequency in Hz. At high frequencies, series resistance becomes negligible. Shunt conductance is also often assumed to be negligible for the dielectric medium in the transmission line.

Consider a simple ETDR setup by operating the transmission line in open-circuit mode. One end of the transmission line is disconnected, while the other end is connected to a pulse-waveform generator and a digital oscilloscope. When the transmission line is intact, the pulse signal travels uninterrupted through the line until it reaches the end before reflecting. With a priori knowledge of the length of the transmission line (*l*_0_) and the pulse wave speed (*v*), the time-of-flight (Δ*t*) for the pulse to travel through the entire intact transmission line and back can be determined. It should be mentioned that the transmission velocity of the incident wave is affected by the type of transmission line, which is approximately 0.6 to 0.9 times the velocity of light [21]. The velocity for any given system can be experimentally determined using an ETDR setup given a fixed *l*_0_ transmission line (with no defects) and by measuring the corresponding Δ*t*:

(2) $$l_{0}=\frac{v∆t}{2}$$

The term *v*Δ*t* in Equation (2) is divided by 2, because the total distance traveled by the incident wave includes its forward direction and its reflected path.

In fact, one of the common uses of ETDR is for localizing discontinuities or breaks in transmission lines. Any discontinuity or damage in the line would induce an impedance mismatch to cause at least a portion of the propagating wave to reflect, which occurs much sooner than when it is undamaged [14]. Similarly, ETDR can also be configured with a built-in sensing element (e.g., in the form of a parallel capacitor, *C′*) as shown in Figure 2b, where stimuli-induced changes in *C′* would cause impedance changes that induce a reflected signal. For these cases, the voltage magnitude of the reflected wave can be quantified by its reflection coefficient (*ρ*). The reflection coefficient is calculated by normalizing the voltage amplitude of the reflected pulse wave (*V_r_*) with respect to the voltage amplitude of the incident pulse wave (*V_i_*) [22]:

(3) $$\rho =\frac{V_{r}}{V_{i}}=\frac{Z_{1}-Z_{0}}{Z_{1}+Z_{0}}$$

where *Z*_0_ is the characteristic impedance of the transmission line, and *Z*_1_ is the characteristic impedance of the discontinuity or mismatch. If the transmission line is intact, then *Z*_1_ *= Z*_0_, the reflection coefficient is 0, which means that no reflected wave would appear. In contrast, a nonzero reflection coefficient (*ρ* ≠ 0) would occur if there is an impedance mismatch (*Z*_1_ ≠ *Z*_0_) anywhere along the transmission line [14]. Thus, the reflection coefficient and the amplitude of the reflected pulse wave can indicate the severity of damage or the response of the sensor (i.e., corresponding to the magnitude of impedance mismatch).

**Figure 3:**
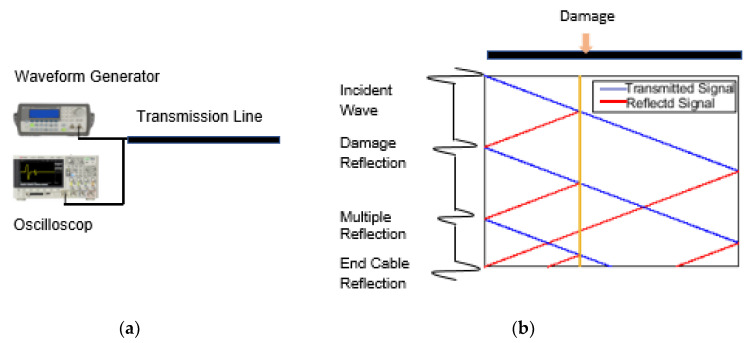
(**a**) The simplest ETDR experimental setup uses a waveform generator and oscilloscope connected to the transmission line. (**b**) The principle of multiple reflections is illustrated.

The simplicity of ETDR, especially when operated in open-circuit mode as shown in Figure 3a, has motivated many researchers to adapt the technique for SHM. In the 1990s, ETDR was successfully used for geotechnical engineering applications, specifically for soil water content measurements [23,24,25]. Since then, ETDR has been used for monitoring civil infrastructure. For example, Miau-Bin [26] used ETDR to detect fracture in concrete structures by monitoring the deformation of a coaxial cable. Lin et al. [27] detected cracks in a photoelastic epoxy specimen using embedded ETDR sensing cables (i.e., RG-174/U coaxial cables). Yankielun et al. [28] showed that ETDR could be used for monitoring bridge scour, which was demonstrated by measuring the length of a probe embedded into soil. Bishop et al. [29] embedded a coaxial cable transmission line and verified crack monitoring in a reinforced concrete structure subjected to shaking table tests. More recently, Lee et al. [14] demonstrated that capacitive nanocomposite sensors could be coupled with ETDR for distributed strain monitoring and crack detection.

Although ETDR has been shown to be very useful, particularly for monitoring the integrity of electrical transmission lines, it is prone to issues such as multiple reflections. ETDR multiple reflections are false positives, where reflected waves are induced and detected but do not correspond to any discontinuity or impedance mismatch in the line [30]. Instead, multiple reflections are the result of the incident and reflected signals traveling simultaneously along the transmission line in both directions, as is illustrated in Figure 3b [30]. In essence, the first true reflected wave would induce additional secondary reflections at the interrogation-measurement location that continue to propagate in the line. The occurrence of multiple reflections is related to the number and locations of impedance mismatches.

In this study, commercial RG-6 coaxial cables were employed as transmission lines for ETDR testing. Capacitive tilt sensors, whose capacitance would vary depending on its tilt angle (see Section 3), were coupled with the transmission line according to the equivalent circuit diagram shown in Figure 2b. Changes in tilt (and thus capacitance) would cause impedance variations that induce a reflected signal, and the voltage amplitude of the reflected signal and reflection coefficient were used to back-calculate the tilt angle at each sensor location. The RG-6 coaxial cables served to carry and transmit the input and reflected pulse signals.

## 3. Capacitive Tilt Sensor Design

### 3.1. Tilt Sensor Design and Operating Principles

A capacitive sensor based on a parallel-plate capacitor setup was designed so that its capacitance would vary linearly with respect to its tilt angle. A benefit of capacitive sensors is their ease in incorporating shielding against ambient electric fields, versus providing electromagnetic interference shielding for other types of sensors [13]. In general, the capacitance of a parallel-plate capacitor (*C_pp_*) can be calculated using:

(4) $$C_{pp}=\frac{\varepsilon A}{d}$$

where ε is dielectric permittivity, *A* is the overlapping area of the conductive parallel plate electrodes, and *d* is the separation distance between the two plates.

**Figure 4:**
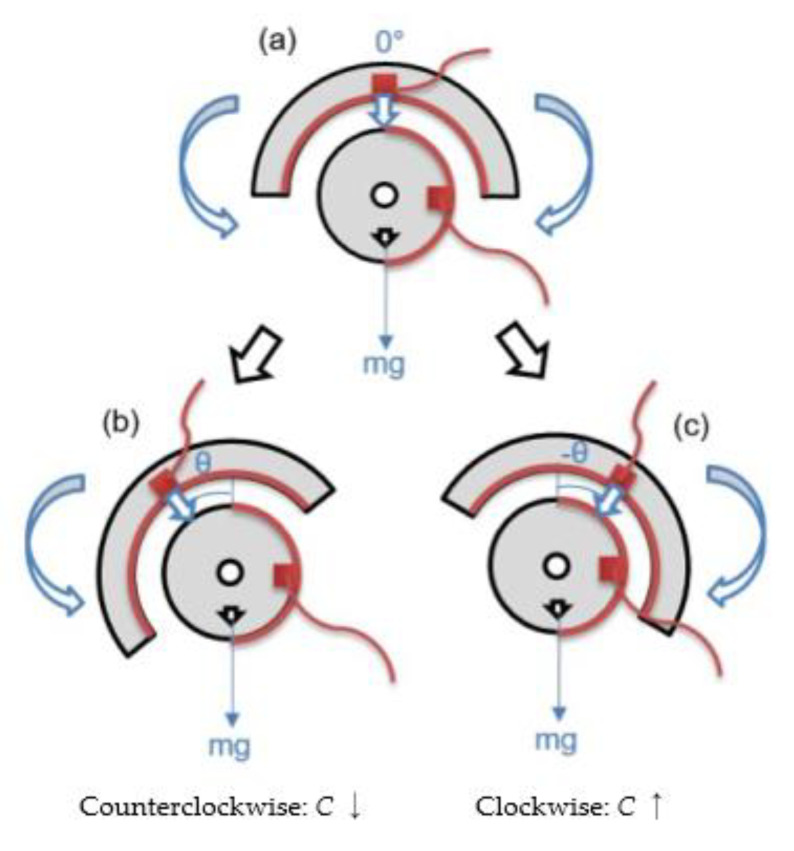
(**a**) The null position of the capacitive tilt sensor, and when it is rotated (**b**) counterclockwise and (**c**) clockwise, are shown.

The design of the capacitive tilt sensor is shown in Figure 4. The sensor consists of two concentrically oriented cylindrical inner and external components, which are completely uncoupled so that each can freely rotate with respect to one another. In Figure 4, the gray area highlights the frame of the sensor, *mg* refers to the total weight of the inner component with respect to its center-of-gravity (CG), the red semicircles are the rectangular parallel-plate electrodes, and the red squares are the electrical connections for the parallel-plate capacitive sensor. The inner component is asymmetrically weighted with a low CG so that the component and its conductive plate remains in the same null configuration regardless of the angle of tilt (*θ*), as is shown in Figure 4a. In contrast, the external component that is affixed to the target structure (e.g., rail track or ties) will tilt or rotate as shown in Figure 4b,c (i.e., in accordance with crosslevel variations).

Figure 4 also shows that, when the sensor rotates counterclockwise, the overlapping area between the parallel conductive plates decreases, thus decreasing capacitance; the opposite is true when the sensor is tilted clockwise. This sensor design enables capacitive *θ* measurements from −90° to 90°, before becoming out-of-range. That said, the angles of tilt that need to be measured for monitoring crosslevel variations are much lower than this range. In fact, the maximum angle of the tilted rail should not be more than 8.2°, according to the track safety standard of the Code of Federal Regulation part 213 [31]. In other words, the dynamic range of the proposed tilt sensor meets the application requirements.

### 3.2. Tilt Sensor Fabrication

**Figure 5:**
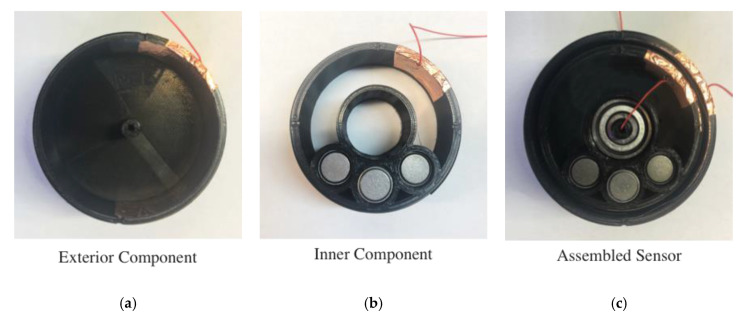
The 3D-printed capacitive tilt sensor consists of an (**a**) external component and (**b**) a concentrically mounted inner component with tungsten weights. (**c**) The uncovered and assembled tilt sensor with ball bearings at the interface is shown.

The inner component, exterior component, and the assembled capacitive tilt sensor are shown in Figure 5. Most of the capacitive tilt sensors were fabricated from polylactic acid (PLA) using a fused deposition modeling Ultimaker 3+ three-dimensional (3D) printer. The external (Figure 5a) and internal (Figure 5b) components of the sensor were printed separately. Component geometries were designed using Autodesk Fusion 360, and the 3D model files (in stl format) were loaded in Ultimaker Cura 3.3 for printing. The entire width of the tilt sensor is 20 mm, while the inner diameter of the external component (Figure 5a) is 50 mm, and the outer diameter of the inner component (Figure 5b) is 47 mm. By mounted the external component and inner component concentrically, the distance between the inner and exterior components is fixed at 1.5 mm. As mentioned earlier, the inner component was designed to be asymmetrically weighted. This was achieved by 3D printing three open cylinders to house Tungsten weights (~70 g), as is shown in Figure 5b. This allowed a significant lowering of the CG of the inner component. 

Upon completion of 3D printing of the inner and exterior components, rectangular conductive copper tape strips were attached to form the parallel-plate capacitor electrodes as shown in Figure 5a,b. A multi-strand electrical wire was soldered to each of the capacitor electrodes and routed outside of the tilt sensor for easy access and for making electrical connections. Within the tilt sensor, polyvinylidene fluoride (PVDF) electrical tape were lined in between the two parallel capacitor electrodes to form the dielectric, as well as for preventing accidental electrical shorting. An NTN 626ZZ ball bearing was also installed at the interface (followed by applying lubricant) between the internal and external components for reducing rotational friction during tilt. Figure 5c shows the uncovered but assembled picture of the capacitive tilt sensor.

## 4. Experimental Details

### 4.1. Characterization of Tilt Sensor Performance

**Figure 6:**
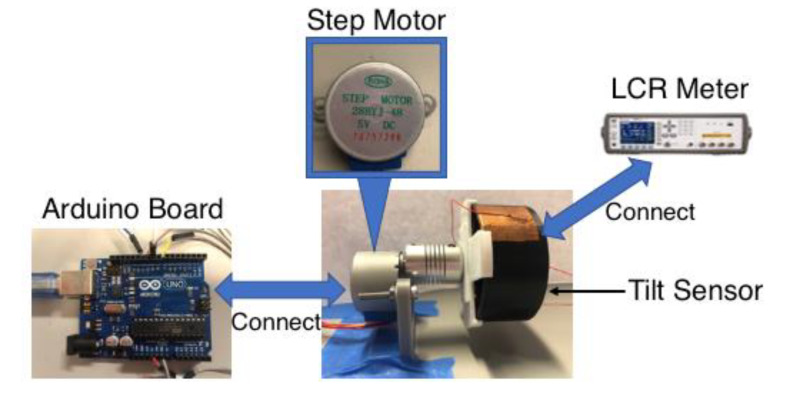
The tilt sensor characterization setup uses an Arduino to control a stepper motor to apply a controlled tilt to the prototype sensor.

The performance of the 3D-printed tilt sensor was characterized by subjecting prototype sensors to different angles of tilt while simultaneously recording their capacitance (Figure 6). Controlled tilt was applied by mounting a tilt sensor to a stepper motor, which was electronically controlled using a programmed Arduino board. The software-controlled motor was commanded to rotate its motor and the tilt sensor in 5° increments so that *θ* changed from −40° to 40°, during which capacitance was recorded using a Keysight E4980A Precision Inductance-Capacitance-Resistance (LCR) Meter. At each level of tilt, ~90 capacitance value were recorded and averaged. Capacitance measurements were obtained using an interrogation signal that was 1 V_p-p_ (peak-to-peak) at a frequency of 100 kHz. For all tests, counterclockwise rotation of the tilt sensor corresponded to a negative angle of tilt, while clockwise rotation was in the positive direction. 

### 4.2. ETDR Multi-Sensor Testing

**Figure 7:**
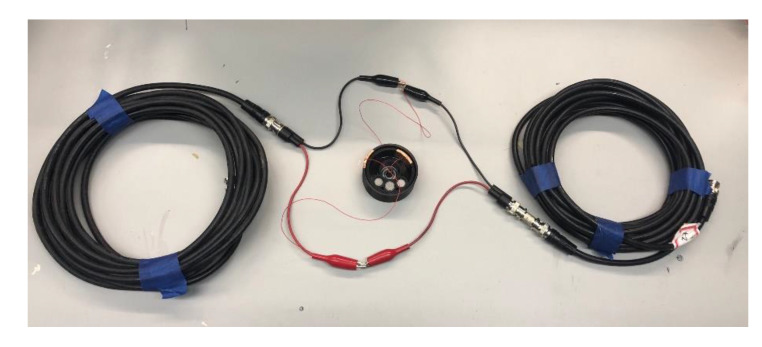
The tilt sensor is connected as part of a transmission line setup using RG-6 coaxial cables.

**Figure 8:**
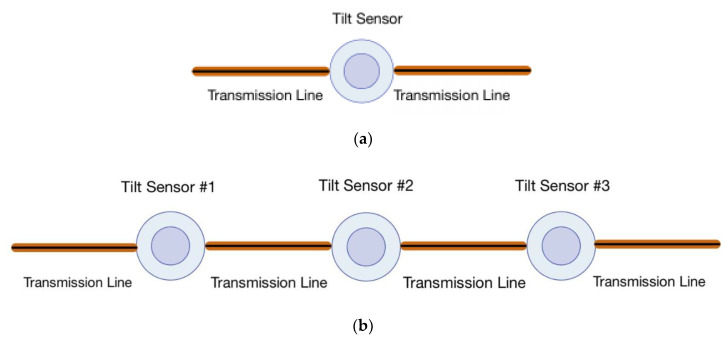
The ETDR test setups consisted of connecting (**a**) a capacitive tilt sensor and (**b**) three tilt sensors in series by 25 ft long cables at opposite ends to form the entire transmission line.

Two sets of tests were conducted to validate the coupling of ETDR with capacitive sensors for tilt monitoring. The tilt sensor was connected in parallel to RG-6 coaxial cable transmission lines using BNC connectors with alligator clips (Figure 7). The first test entailed connecting two transmission lines, each 25 ft (7.62 m) long, to a 3D-printed capacitive tilt sensor, as illustrated in Figure 8a. The second test employed three capacitive tilt sensors, which were each connected by 25 ft (7.62 m) transmission lines as shown in Figure 8b. For both test setups, an Agilent 33600A Series waveform generator was connected at one end to inject a half-cycle, 10 V_p-p_, 8 ns pulse width, sinusoidal signal to interrogate the transmission line. Reflected signals were recorded using a Keysight DSOX3024T digital oscilloscope (with a maximum sampling rate of 5 GSa/s and bandwidth of 200 MHz) connected at the same end as the waveform generator. Meanwhile, each capacitive tilt sensor was also connected to a stepper motor similar to the setup described in Section 4.1. Different angles of tilt ranging from −40° to 40° were applied to the sensors, while the ETDR system simultaneously interrogated the sensors and recorded the reflected wave response. In addition, the capacitance of each tilt sensor for each angle of tilt case was also measured using a Keysight E4980A LCR meter. All the tests were conducted at room temperature of ~23 °C.

## 5. Results and Discussion 

### 5.1. Capacitive Tilt Sensor Characterization Results

**Figure 9:**
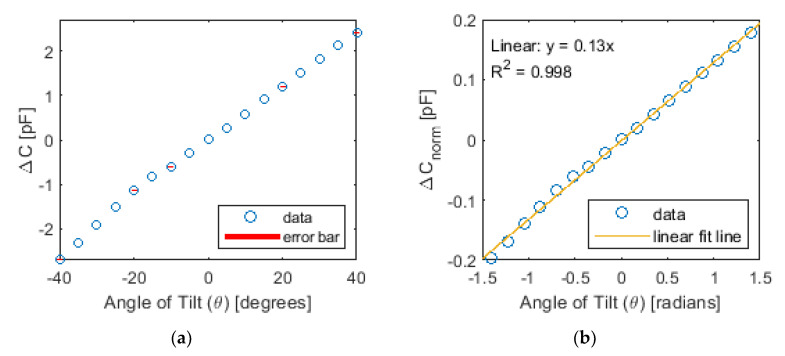
(**a**) The capacitance response of the sensor subjected to different angles of tilt and (**b**) the corresponding normalized change in capacitance data with a linear best-fit line are plotted.

As mentioned in Section 4.1, the 3D-printed sensors were subjected to controlled tilt tests, while their capacitance was measured using an LCR meter. Figure 9a plots a representative result of the measured capacitance with respect to the angle of tilt. Each data point corresponds to the average change in capacitance, while the error bars are the standard deviations of repeated measurements. The error for each data point is too small, so many error bars are barely visible (some can be seen in Figure 9a). Figure 9b plots the normalized change in capacitance (∆*C_norm_*) of the same dataset as a function of the angle of tilt (in radians). Normalized change in capacitance was calculated by:

(5) $$∆C_{norm}=\frac{∆C}{C_{0}}=\frac{C_{i}-C_{0}}{C_{0}}$$

where *C*_0_ is the capacitance when the sensor was in its null position where the angle of tilt is *θ*_0_ = 0 rad, and *C_i_* is the capacitance corresponding to an angle of tilt of *θ_i_*. A linear least-squares regression line was fitted to the data in Figure 9b. Its coefficient of determination, or *R*^2^, was found to be ~0.998, thus indicating strong linear sensing performance. Furthermore, the sensitivity (*S*) of the sensor is equivalent to the slope of the linear best-fit line shown in Figure 9b, which can be calculated using:

(6) $$S=\frac{∆C_{n}}{∆\theta }=\frac{C_{i}-C_{0}}{\theta_{i}-\theta_{0}}$$

For the various prototype capacitive tilt sensors fabricated, the average sensitivity was estimated to be ~0.13. From the results obtained, the calibration curve for these capacitive tilt sensors is:

(7) $$∆C_{norm}=0.13\left(∆\theta \right)$$

By considering the maximum deviation (or residuals) among all the measured data in Figure 9, one can also conservatively estimate the accuracy of the sensor to be ~±0.16°, which is only ~2% of the maximum angle of the tilted rail as specified in the track safety standard of the Code of Federal Regulation part 213 [31]. Overall, the results presented in Figure 9 successfully validated the tilt sensing capabilities of the proposed 3D-printed sensor.

### 5.2. ETDR with a Single Tilt Sensor 

**Figure 10:**
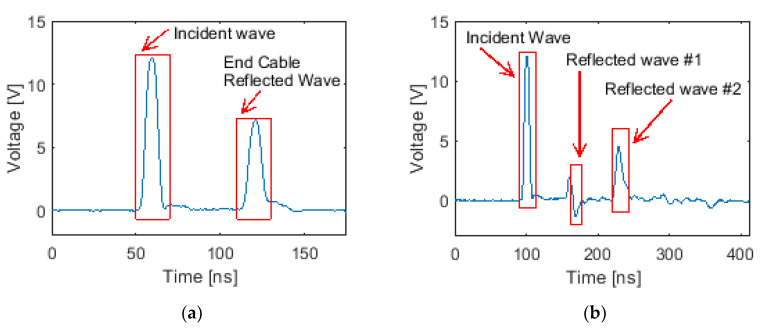
(**a**) ETDR testing of an RG-6 coaxial cable transmission line in open-circuit mode was performed to determine the pulse speed. (**b**) ETDR tests with a single capacitive tilt sensor was performed, and a representative voltage-time history response is plotted. The reflected waves are marked accordingly in each plot.

Section 4.2 outlined the details of the experimental setup for the ETDR tests. Prior to testing, the electrical pulse wave speed was determined by injecting the same waveform in a 25 ft (7.62 m) transmission line. The transmission line was operated in open-circuit mode and without any capacitive tilt sensors. Figure 10a plots a representative voltage-time history response as recorded by the digital oscilloscope. The first pulse waveform identified in Figure 10a corresponds to the incident signal generated by the waveform generator. This pulse was launched into the transmission line at one end of the transmission line and reflected back when the wave reached the other end of the transmission line [32]. The second waveform identified in Figure 10a corresponds to the reflected wave due to open-circuit conditions. The time interval between the incident wave and the reflected wave was ~63 ns. An accurate calculation of the time interval was determined by comparing the difference in times between the peak of the incident wave with the peak of the reflected wave. Then, using Equation (2), the transmission velocity was calculated to be 2.419 × 10^8^ m/s, which is ~81% of the speed of light. This value is consistent with the standard propagation velocity of commercial RG-6 coaxial cables, which has been reported to be 82% of the speed light [33].

Upon determining the pulse wave speed, the ETDR sensing characterization test involving a single tilt sensor, as shown in Figure 8a, was conducted. With the introduction of the capacitive tilt sensor in the transmission line, an impedance mismatch was created, and a portion of the energy of the incident wave would be reflected. Because the sensor was at a fixed position in the transmission line, the time-of-flight of the reflected wave remained the same for all angle-of-tilt test cases. Instead, capacitance change of the tilt sensor would induce a corresponding change in the voltage amplitude of the reflected wave due to impedance variations. Figure 10b plots a voltage-time history recorded for the case when the sensor was tilted to 25°. Reflected wave #1 was due to the impedance mismatch between the tilt sensor and the transmission line. Reflected wave #1 was also a negative pulse due to the decrease in impedance introduced by the tilt sensor. Equation (1) indicates that impedance decreases with the square root of increasing shunt capacitance (as compared to the transmission line). The positive pulse right before Reflected wave #1 in Figure 10b might be caused by the use of BNC-alligator clips. Regardless, the remaining signal was transmitted through the transmission line, and Reflected wave #2 was the reflection of the remnant waveform off the end of the transmission line, which was operated in open-circuit mode.

The time-of-flight of the reflected waves was also investigated. The time interval between the incident wave and Reflected wave #1 was 65 ns, and the time interval between Reflected wave #1 and Reflected wave #2 was 61 ns. The distance between the start of the transmission line and the location of the tilt sensor, as well as the distance from the tilt sensor to the end of the open-circuit transmission line, were calculated as 25.8 ft (7.84 m) and 24.2 ft (7.38 m), respectively. These length estimates of each transmission line segment matched closely with the theoretical value, which was 25 ft or 7.62 m. Thus, these results successfully validated that ETDR could be used for interrogating capacitive tilt sensors incorporated in transmission lines. Furthermore, time-of-flight measurements correctly identified the location of the tilt sensor.

**Figure 11:**
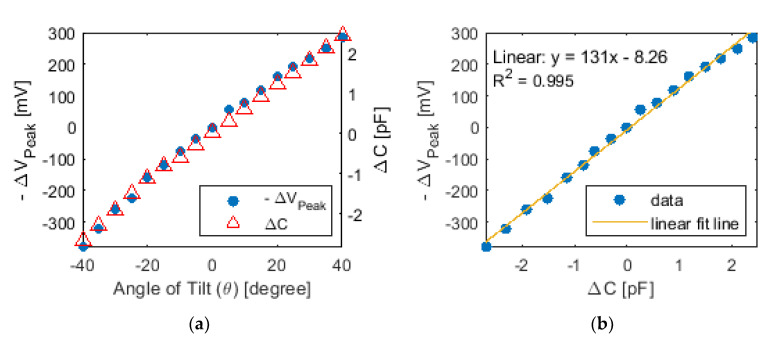
(**a**) The negative change in peak voltages of Reflected wave #1 from ETDR tests and the capacitance change of the tilt sensor are overlaid and plotted with respect to the angle of tilt. (**b**) Replotting the same set of data with its linear best-fit line confirmed linear tilt sensing performance.

After repeating this test for different angles of tilt, the recorded voltage-time histories could be processed to identify Reflected wave #1 and to extract its peak voltage amplitude (Figure 11). The negative of voltage amplitude with respect to its null position (0° case) was plotted as a function of the angle of tilt in Figure 11a. In addition, the capacitance of the tilt sensor was also measured using the Keysight LCR meter during testing. The change in capacitance of each angle-of-tilt test case with respect to its null position (0° case) is also overlaid in Figure 11a. These results indicated that the intensity of the peak voltage of the reflected wave increased in tandem with increasing (from negative to positive) angles of tilt and the sensor’s capacitance. This finding is consistent with the reflection coefficient or *ρ*, where Equations (1) and (3) indicate that *ρ* becomes more negative with decreasing impedance (i.e., increasing capacitance) of the tilt sensor. Larger absolute values of *ρ* mean that the voltage amplitude of Reflected wave #1 would increase as the sensor was tilted more clockwise, and its capacitance increased. The value of *ρ* is negative due to the smaller impedance of the tilt sensor as compared to the impedance of the unit length of the transmission line. Overall, strong linear sensing performance was again confirmed by fitting a linear least-squares regression line to the data, as shown in Figure 11b, and *R*^2^ was 0.995. The results in Figure 11 validated tilt monitoring using an ETDR test setup.

### 5.3. ETDR with Multiple Tilt Sensors

**Figure 12:**
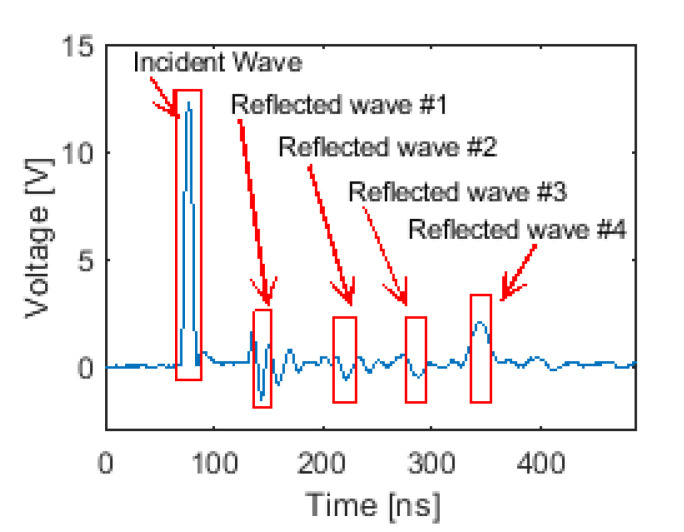
A representative voltage-time history of ETDR testing of a transmission line with three capacitive tilt sensors connected in parallel and in an open-circuit configuration is shown.

One of the main benefits of ETDR is its ability to interrogate multiple electrical circuit elements and/or sensors for damage along its entire transmission line [20]. Therefore, to validate using ETDR for interrogating multiple tilt sensors with a single excitation signal, tests were performed using three capacitive tilt sensors incorporated in a transmission line (Section 4.2 and Figure 8b). Figure 12 plots a representative voltage-time history response, which shows the incident wave (similar to before), as well as multiple reflected waves, due to the three capacitive tilt sensors. Confirmation of these identified waveforms was determined by calculating their respective positions in the transmission line using times-of-flight extracted from the peak of each reflected wave. Similar to Section 5.2, Equation (2) was used, and their locations in the transmission line were found to be 25.8 ft (7.84 m), 53.5 ft (16.31 m), and 78.9 ft (24.05 m). In addition, Reflected wave #4 was due to the incident wave reflecting from the open-circuit end of the transmission line, and its position was 106.6 ft (32.5 m). These experimental results matched closely with their actual positions of 25 ft (7.62 m), 50 ft (15.2 m), 75 ft (22.9 m), and 100 ft (30.5 m).

**Figure 13:**
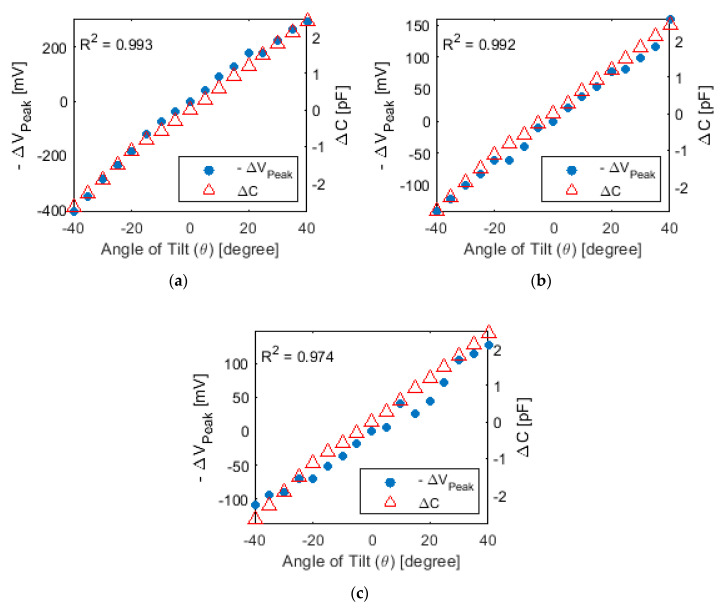
ETDR testing was performed using three sensors in a transmission line. The change in peak voltages of tilt sensors (**a**) #1, (**b**) #2, and (**c**) #3 for different angles of tilt are plotted. The corresponding sensor’s change in capacitance results are also overlaid in each plot.

Next, ETDR testing proceeded with assigning a specific angle of tilt to one of the 3D-printed sensors while fixing other tilt sensors in the null position. Different conditions were tested, and a representative set of results is plotted in Figure 13. Overall, the same observations can be seen in Figure 13, as in the case of a single capacitive sensor in Figure 11a. The negative value of change in peak voltages for each reflected wave increased in tandem with increasing clockwise tilt and change in capacitance. All three sensors also exhibited a linear relationship between voltage amplitude and angle of tilt. The *R*^2^ values for Tilt Sensors #1, #2, and #3 (according to Figure 13) were 0.993, 0.992, and 0.974, respectively, which again confirmed their linear tilt sensing properties.

However, based on these *R*^2^ values and from Figure 13, the trend shows that tilt sensing linearity slightly deteriorated for each additional capacitive sensor added to the transmission line. Several sources of error could contribute to this observation. First, the amplitude of the reflected wave was significantly reduced for each added sensor and became harder to characterize, which was due to both signal attenuation and only a portion of the input pulse energy passing through each sensor. Second, constructive and destructive interference could have occurred and disrupted the characteristic shape of the waveform, as is evident in Figure 12 (for Reflected waves #3 and #4). Last, the reduction in voltage amplitude made these signals more susceptible to noise, which reduced the accuracy of identifying the true peak voltage amplitude. These limitations suggest that there may be a theoretical maximum for the total number of tilt sensors that can be incorporated in a given transmission line (i.e., for any given ETDR hardware setup). The amplitudes of the reflected wave of the first, second, and third ETDR sensor when the tilt sensor was at its null position (0° case) were approximately −2 V, −1 V, and −0.6 V, respectively. By observing the trend of the decrease in peak voltage amplitude, it can be estimated that, using this study’s test setup, a maximum of eight tilt sensors can be connected and interrogated in a transmission line. Methods to circumvent these issues (e.g., to enhance the reflected waveforms or to include more sensors) include using a higher-amplitude voltage input pulse signal and/or employing signal processing and filtering techniques. Overall, the results confirmed that ETDR was successfully used for simultaneously interrogating multiple capacitive tilt sensors in a single transmission line.

## 6. Conclusions

In this study, a new, capacitive, railroad crosslevel tilt sensor was designed and fabricated by 3D-printing, which was then coupled with ETDR. As compared to many conventional SHM systems that require multiple sensing channels to acquire data from its sensors, ETDR is unique in that only a single data acquisition channel is needed. Sensors are connected in parallel in a transmission line, and an electrical pulse is injected to interrogate all the sensors in the line, while the recorded voltage-time history captures the responses of all sensors. This study began with characterizing the capacitive sensing performance of 3D-printed prototype tilt sensors. The results showed that the sensor’s capacitance varied linearly with respect to its angle of tilt. In addition, a conservative estimate of its accuracy was found to be ±0.16°. Then, a capacitive tilt sensor was incorporated in a transmission line and subjected to ETDR testing, while the sensor was subjected to different angles of tilt. Initial tests confirmed that the time-of-flight measurements correctly identified the location of the sensor in the line. The peak voltage amplitudes of the reflected wave also varied linearly according to the angle of tilt. Last, the interrogation of multiple tilt sensors using ETDR was also successfully validated. The results showed that the angles of tilt of all three sensors were reliably measured, although sensing performance slightly deteriorated with each additional sensor connected to the transmission line. Future research will focus on improving the accuracy of the tilt sensor and demonstrating the use of more sensors in a single transmission line. Laboratory tests involving a scaled railroad track are also planned.
